# Efficacy of sleep promotion strategies in childcare settings and sleep outcomes in young children: a systematic review

**DOI:** 10.3389/frsle.2026.1818620

**Published:** 2026-07-22

**Authors:** Belinda O'Hagan, Sumner Chmielewski, Naomi Deokule, Alexandra Josephson, Tayla Von Ash

**Affiliations:** Department of Behavioral and Social Sciences, Brown University School of Public Health, Providence, RI, United States

**Keywords:** childcare, nap, preschool, sleep, systematic review

## Abstract

**Objectives:**

To summarize findings and provide methodological critique of studies examining the impact of sleep promotion strategies in childcare on young children's sleep outcomes.

**Methods:**

The search run in PubMed, PsycINFO, and CINAHL yielded a total of 3,528 unique articles. Eligible studies included young children (under 5 years old); assessed the impact of at least one sleep promotion strategy whether structural (e.g., mandatory naptime) or behavioral (e.g., music) with both within- and between-subject designs eligible); and included at least one quantitative child sleep outcome.

**Results:**

6 studies met the eligibility criteria. Identified childcare sleep promotion strategies included mandatory naptime (*n* = 4), music (*n* = 1), and massage therapy (*n* = 1). Findings indicated that mandatory naptime was associated with increased napping. One study found that mandatory naptime was associated with reduced nighttime sleep and total daily sleep duration. Music and massage therapy were associated with reduced nap latency.

**Conclusions:**

There is a paucity of research on sleep promotion strategies in childcare. This is an important research gap given the established health benefits of sufficient sleep and that many children from historically disadvantaged backgrounds depend on naps to meet their sleep needs. Future studies examining the effects of sleep promotion strategies other than mandated naptime, such as the sleep environment and provider behaviors, are needed.

## 1 Sleep promotion strategies in childcare settings and sleep outcomes in young children: a systematic review

There is ample evidence about the importance of adequate sleep during early childhood (Chaput et al., 2017; Matricciani et al., 2019; Reynaud et al., 2018) including associations with positive cognitive outcomes (Cai et al., 2023; Vaughn et al., 2015), body composition (Taveras et al., 2008), emotional wellbeing (Chaput et al., 2017), and social outcomes (Vaughn et al., 2015). The American Academy of Sleep Medicine recommends that preschoolers (3–5 years) sleep 10–13 h per day including naps to promote optimal health (Paruthi et al., 2016). However, many children are not getting sufficient sleep and past studies have documented the presence of sleep disparities during early childhood based on race and ethnicity with children identifying as Black, Hispanic, and Asian less likely to meet sleep recommendations than non-Hispanic white children (Ahn et al., 2021; Lupini and Williamson, 2023; Nevarez et al., 2010). Related, prior research has shown that non-white children have shorter nighttime sleep durations but longer daytime sleep durations compared to non-Hispanic white children (Crosby et al., 2005; Nevarez et al., 2010; Zhang et al., 2021). Therefore, racial and ethnic minoritized children may be more dependent than their non-Hispanic white counterparts on daytime sleep to meet sleep (Crosby et al., 2005; Nevarez et al., 2010; Zhang et al., 2021). Creating environments conducive to daytime sleep is thus important for child health and development and may be especially important for racial and ethnic minoritized children.

Childcare has become an essential service for families (National Association for the Education of Young Children, 2021). In the United States, approximately 59% of children under the age of five and not in kindergarten spend time in childcare (U.S. Department of Education, and National Center for Education Statistics, 2021) and it is estimated that children spend a quarter of their time in childcare settings sleeping (Tandon et al., 2015). Prior research demonstrates the efficacy of caregiver initiated sleep promotion strategies targeting young children with a focus on home-based sleep parenting interventions (Agaronov et al., 2018; Albakri et al., 2021; Magee et al., 2022). However, little is known about the efficacy of sleep promotion strategies in childcare settings, even though most children regularly nap in this context (von Ash et al., 2025). Importantly, prior research shows that children's sleep behavior is influenced by the type of childcare they receive suggesting variability in aspects of care related to sleep across different types of childcare settings (von Ash et al., 2025). At the same time, studies have also demonstrated variability in nap environments and nap related provider practices across sites of the same childcare type (O'Hagan et al., 2025; Staton et al., 2016; von Ash et al., 2021).

Qualitative work has shown that while childcare providers generally have the same goal of getting children to rest, there are variations around ideas on the best ways to achieve this goal and whether the expectation is that children actually sleep vs. rest their bodies during nap time (O'Hagan et al., 2025). Some childcare providers view helping children fall asleep (e.g., rubbing their backs) as beneficial whereas others view this practice as hindering children's ability to self-soothe (O'Hagan et al., 2025). Meanwhile, nap time is often a time when providers need to tend to other responsibilities (e.g., cleaning, curriculum planning) and take their lunch break. Child to child variation in sleep needs adds further complexity to the way childcare providers must navigate nap time. To our knowledge, comprehensive effective childcare-based sleep interventions have not yet been developed likely because evidence-based recommendations for childcare providers are lacking (McGuire, 2012). Notably, despite considerable growth in the literature on sleep in childcare over the past decade, interventions and practical recommendations for childcare providers remains limited.

Best practices for promoting sleep at home and during childcare are likely similar. However, there are differences between the two settings that could not only pose as barriers to implementing best practices but also impact their efficacy. The ratio of children to caregivers in childcare for example, as well as other logistics, can make it difficult to implement sleep schedules based on each individual child's needs. Thus, sleep environments and routines likely differ for children while in childcare necessitating the need for research to identify effective sleep promotion strategies for childcare settings. Indeed, a recent systematic review, which summarized associations between childcare attendance, type of arrangement, and time spent in care with child sleep outcomes, concluded that more research is needed to develop interventions and inform best practices for sleep promotion in childcare (von Ash et al., 2025). In response, the present systematic review aims to examine the efficacy of sleep promotion strategies in childcare on children's sleep outcomes. For the purposes of this review, we define sleep promotion strategies as actions or policies that childcare providers enact to enable or encourage child sleep (e.g., carving out time during the day specifically for rest, helping children rest by rubbing their backs).

## 2 Methods

This review used the same search strategy as another systematic review conducted by our team to examine associations between childcare attendance, arrangement type, and dose with child sleep outcomes (von Ash et al., 2025). The protocol was registered in the PROSPERO database (CRD42024508992). In consultation with a health sciences librarian, a single search strategy was deemed appropriate for both reviews given that in attempt to minimize missing any relevant literature for the first review, the search strategy consisted of just two search strings: one focused on sleep and the other on childcare. These search strings were deemed adequate to capture all relevant literature pertaining to the research question for the current review (i.e., examining the efficacy of previously tested sleep promotion strategies within the childcare setting).

### 2.1 Search strategy

**Figure 1:**
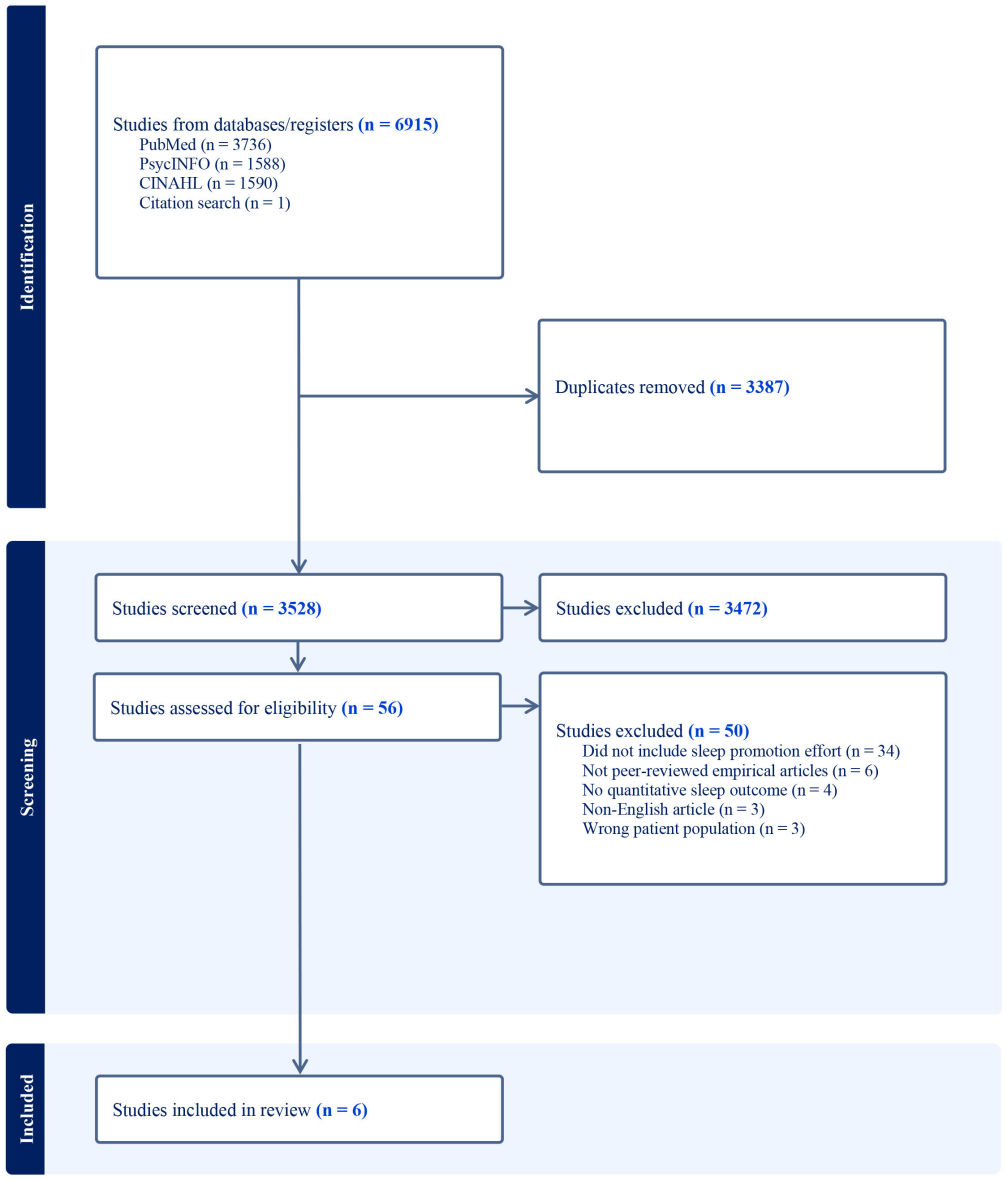
PRISMA flowchart.

This review was conducted in accordance with the Preferred Reporting Items for Systematic Reviews and Meta-analyses (PRISMA) methodology (Moher et al., 2009) (Figure 1). The search was conducted by a health sciences librarian in March 2024. Three databases were used, specifically PubMed, PsycINFO, and CINAHL. Keywords, including Medical Subject Headings (MeSH), related to “sleep” and “childcare” were used. Only English articles were included. There was no restriction posed on publication year and geographical location. The full search strategy has been previously published (von Ash et al., 2025).

### 2.2 Eligibility criteria

The following criteria were used: included participants <5 years old, assessed the efficacy of at least one sleep promotion strategy in childcare (e.g., naptime protocol, provider behavior, sleep environment) with both within- and between-subject designs eligible, and included at least one sleep outcome (e.g., duration, latency). Sleep promotion strategy is defined as any action that providers take to promote sleep during childcare hours. Because we anticipated there were few published studies of sleep promotion strategies in childcare, any type of sleep outcome was acceptable so long as it was quantitative. Because the age in which children start school and exit childcare may differ between countries, studies whose participants' maximum age was older than five were also included if they also included participants younger than 5 years old. No restrictions were posed in terms of study location, design, and publication year.

### 2.3 Screening process

Identified articles were imported to Covidence, an online system to manage systematic reviews (Covidence, n.d.). The first step of the screening process was at the title and abstract level. Articles that were deemed to meet the inclusion criteria were screened for a second time at the full text level. The inclusion criteria were again used to determine whether articles were moved to the final extraction step. At both levels, screenings were conducted independently by the first author and at least one co-author, with disagreements resolved by the senior author. Additionally, we conducted a manual citation search on all articles meeting our eligibility criteria, with references screened by the first author at the title and abstract level and both the first and senior author at the full-text level. Percent agreement across reviewer pairs ranged from 74–95% at the title and abstract level, 58–79% at the full-text level, and was 95% for data extraction.

### 2.4 Data extraction and quality appraisal

The first and senior authors independently extracted data. Disagreements were discussed until consensus was reached. The following data were extracted: first author's last name, publication year, site number, child sample size in the final analysis, age range, country, percentage of females, racial or ethnic composition, study design, sleep promotion strategy assessed, covariates adjusted for in the analyses, and measured sleep outcomes.

The Quality Assessment Tool for Observational Cohort and Cross-sectional Studies and the Quality Assessment Tool of Controlled Intervention Studies were used to assess the quality of included studies (National Heart, Lung, and Blood Institute, 2021). Both tools consist of 14 items that could be rated as “yes,” “no,” “not applicable,” “not reported,” or “cannot determine,” with items rated as “yes” being assigned to one point. Consistent with prior research, studies were given a rating of good if tallied scores were between 10 and 14 points, fair if tallied scores between 5 and 9 points, and poor if tallied scores were between 0 and 4 points (DeFrancisis et al., 2025; Kamara et al., 2018). The first and senior authors independently assessed the study quality and met until a consensus on discrepancies in scoring were resolved.

## 3 Results

### 3.1 Search results

The database searches yielded the following articles: 3,736 from PubMed, 1,588 from PsycINFO, and 1,590 from CINAHL (Figure 1). Out of these articles, 56 were deemed eligible for full-text screening. Six studies out of 56 met all the eligibility criteria.

### 3.2 Characteristics of included studies

The six included studies were published between 1996 and 2022 (Field et al., 1996; Field, 1999; Fujii et al., 2022; Staton et al., 2015, 2017; Thorpe et al., 2018). Combined, the studies involved 2,410 participants with sample sizes varying from *n* = 24 to *n* = 2,114. There was also large variation in the number of sites, ranging from single site studies to 130 sites. Studies had either two (i.e., intervention vs. no intervention) or three (i.e., intervention with differing doses) comparison conditions. Data were collected in three countries namely Australia (*n* = 3), United States (*n* = 2), and Malaysia (*n* = 1). Four studies were observational [with three using a cross-sectional design (Fujii et al., 2022; Staton et al., 2017; Thorpe et al., 2018) and one using a longitudinal design (Staton et al., 2015)], while two were experimental (cross-over and waitlist-control designs) (Field, 1999; Field et al., 1996).

Most studies (*n* = 4) targeted children 3 years and older. One study also included 2-year-olds, while the last targeted 1–2-year-olds; the search yielded no studies testing sleep promotion strategies in childcare on infants. The examined sleep promotion strategy in four of the six studies included making naps mandatory with varying durations assessed across studies (Fujii et al., 2022; Staton et al., 2015, 2017; Thorpe et al., 2018). The other two studies, which used an experimental design, examined the use of music and massage therapy as sleep promotion strategies (Field, 1999; Field et al., 1996).

Assessed sleep outcomes across studies included latency (i.e., the amount of time it took children to fall asleep during nap time) (Field, 1999; Field et al., 1996), timing (i.e., what time children started napping, ended napping, went to bed at night, and woke in the morning) (Fujii et al., 2022), duration (i.e., how long children napped, slept at night, and slept across 24 h) (Fujii et al., 2022; Staton et al., 2015), nap frequency (i.e., how many days per week children napped) (Fujii et al., 2022; Staton et al., 2015), and proportion of children who napped (i.e., how many children of all the children in attendance took a nap on a given day) (Staton et al., 2017; Thorpe et al., 2018). Sleep was measured either by observation or caregiver report (either parents or childcare providers). Observation was more common (Field, 1999; Field et al., 1996; Staton et al., 2015, 2017; Thorpe et al., 2018) compared to caregiver report (Fujii et al., 2022; S. L. Staton et al., 2015). All studies were rated as “fair” using the aforementioned quality assessment methods (National Heart, Lung, and Blood Institute, 2021) (Table 1).

**Table 1:** Sample and study characteristics (*n* = 6).

First author (year)	Sample characteristics						Study characteristics			Summary of Findings
	Site or classroom sample size	Child sample size	Age range	Country	% female	Racial or ethnic composition	Study design and comparison conditions	Covariates adjusted for	Sleep outcome(s)	
Field et al. (1996)	Not specified	28	2–4 years	United States	50%	NR	Experimental (waitlist control), 20-min massage therapy 2 x a week for 5 weeks; no massage (waitlist control)	Age	Latency to sleep	Latency to sleep decreased from the 1st day to the last day of massage therapy for children assigned to the massage therapy group, but not for children assigned to the control group.
Field (1999)	2 (each examined for 2 days with music and 2 days without music)	24	1–2 years	United States	NR	58% Caucasian, 22% Hispanic, 17% African American and 3% other	Experimental (cross-over), 2 days with music, 2 days without music	None	Latency to sleep	Latency to sleep was shorter following when children had music vs. not. The effect was stronger for toddlers compared to preschoolers.
Fujii et al. (2022)	4 (3 with optional naps, 1 with mandatory naps)	33	3–6 years	Malaysia	52%	100% Malay ethnicity	Cross-sectional, optional nap (full day private kindergarten) vs. mandatory nap (half day public kindergarten and then childcare	Weekday vs. weekend (for comparisons between type of care)	Bedtime, wake time, nap start time, nap end time, duration (nap, nighttime, and 24-h), napping frequency)	Children with mandatory naps had longer 24-h sleep durations on weekdays and a shorter 24-h sleep durations on weekends compared to those with optional naps The proportion of children who took a nap was higher among those with mandatory naps compared to those with optional naps overall and on weekdays but lower on weekends. However, there were no apparent tests to examine if these differences were significant. Null findings for bedtime, wake time, nap start time, and nap end time.
Staton et al. (2015) ^*^	130 center-based classrooms	168	4–6 years	Australia	45%	NR	Longitudinal (assessed at baseline and 12 months), sites with > 60 min of mandatory naptime vs. 0-60 min of mandatory naptime	Age, gender, temperament, family income, parental education, days/week childcare, childcare quality, program type	Napping frequency; duration (nap, nighttime, and 24-h)	Children in childcare sites that mandate*d* > 60 min of naptime were more frequent nappers at baseline and had longer nap durations than those in sites that mandated 0-60 min of nap at both baseline and 12 months. They also had shorter nighttime sleep both at baseline and 12 months later. Additionally, while there was no group difference in total sleep duration at baseline, they had shorter total sleep duration at 12 months.
Staton et al. (2017) ^*^	113 (24 with < 30 min. nap, 41 with 31–60 min. nap, 48 with >60 min. nap)	2,114	3–6 years	Australia	NR	NR	Cross-sectional, ≤ 30 min. naptime vs. 31–60 min. naptime vs. > 60 min, naptime	Age range, nap start time, emotional support, socioeconomic status, program type	Number and proportion of children napping	There was a significant dose response relationship between mandatory naptime length and the number and proportion of children napping, with longer naptimes being associated with a higher incidence in napping.
Thorpe et al. (2018)	6 (4 mandatory naptime, 2 flexible)	43	3–4 years	Australia	53.5%	NR	Cross-sectional, classrooms with mandatory naptime (no alternative activities allowed) vs. flexible naptime (allowed for alternative activities)	None	Proportion of children napping	A higher proportion of children napped in sites with mandatory naptime policies (43%) compared to those with flexible naptime policies (2%), though no measure of statistical significance was included.

NR: Not reported. ^*^(Staton et al. 2015) and (Staton et al. 2017) shared overlapping samples from the same research group, which could affect the independence of findings from the two studies.

### 3.3 Study findings summary

The tested behavioral sleep promotion strategies were associated with children falling asleep faster during childcare hours. Two studies involved behavioral sleep promotion strategies, namely music and massage therapy (Field, 1999; Field et al., 1996). Notably, both were written by the same primary author (Field, 1999; Field et al., 1996). Both used an experimental design (i.e., cross-over and waitlist control) and compared daytime sleep latency when the strategy was present vs. not (Field, 1999; Field et al., 1996). In the 1996 study, the reported mean age of participants was 39 months (3.25 years) old (Field et al., 1996). Participants received 20-min massage sessions twice a week over 5 weeks for a total of 10 sessions (Field et al., 1996). In the 1999 study, the reported mean age was 19 months (1.6 years) for the toddler group and 32 months (2.7 years) for the preschool group (Field, 1999). Classical guitar music by Christopher Parkening was played for two non-consecutive days over the course of 4 days (i.e., music played on days one and three in the toddler classroom only and on days two and four in the preschool classroom only) (Field, 1999). In both studies, the researchers found there was a reduction in daytime sleep latency after introducing the strategy (Field, 1999; Field et al., 1996).

In addition, mandated naps during childcare hours increased the likelihood of children falling asleep. The remaining four studies examined the effects of requiring naptime for preschool age children (3–6 years old) (Fujii et al., 2022; Staton et al., 2015, 2017; Thorpe et al., 2018). Two studies compared sleep outcomes in sites with mandatory vs. flexible naptime (Fujii et al., 2022; Thorpe et al., 2018). Three out of the four studies defined mandatory naptime as a period of time during childcare during which children are required to rest and not allowed to engage in other activities (Staton et al., 2015, 2017; Thorpe et al., 2018). Fujii et al. (2022) however did not elaborate on how mandatory nap was operationalized beyond a common start time (i.e., 2 PM). Fujii et al. (2022) found that total daily sleep duration was longer on weekdays for children with mandatory naptime compared to children with optional naptime, but shorter on weekends. No differences were found in terms of nighttime sleep duration, bedtime, wake time, nap start time, or nap end time (Fujii et al., 2022). However, the proportion of children who napped was higher in sites with mandatory naptime compared to sites with optional naptime (Fujii et al., 2022). Higher proportion of napping children in sites with mandatory naptime compared to sites with flexible naptime was also reported by Thorpe et al. (2018), though, in both studies, no measure of statistical significance was included for the between-group difference in proportion of napping children (Fujii et al., 2022; Thorpe et al., 2018). The absence of statistical significance testing limited the extent to which findings from these studies could be interpreted.

Lastly, longer mandatory naptimes were also associated with higher incidence of napping. The other two studies examined mandatory naptime in terms of dosage (Staton et al., 2015, 2017). Staton et al. (2015) measured sleep outcomes in sites with 0–60 min of mandatory naptime vs. sites with >60 min of mandatory naptime at two time points 12 months apart (Staton et al., 2015). At baseline, children in sites that mandated >60 min of nap napped more frequently compared to children in sites that mandated < 60 min of naptime (Staton et al., 2015). At both baseline and follow up, children in sites that mandated >60 min of naptime had shorter nighttime sleep durations compared to children in sites that mandated < 60 min of naptime (Staton et al., 2015). Furthermore, although there was no group difference in total daily sleep duration at baseline, children in sites that mandated >60 min of naptime had shorter total daily sleep durations at 12 months (Staton et al., 2015). Similar findings were reported by Staton et al. (2017) when comparing the sleep outcomes of children in sites with ≤ 30 min, 31–60 min, and >60 min of mandated naptime (Staton et al., 2017). The authors found a significant dose response relationship in mandatory naptime duration and the number and proportion of children napping, with longer mandated naptime being associated with a higher incidence of napping (Staton et al., 2017). As both studies involved overlapping samples, findings should be interpreted with caution.

Taken as a whole, findings across studies suggest that modifiable aspects of how childcare providers approach naptime impact child sleep. Findings were consistent that having mandatory naptime and longer periods of it increased the likelihood of children napping. Moreover, helping children fall asleep by playing music and providing massages decreased the time it took for them to fall asleep. However, synthesis across studies is limited given the paucity of studies, including that two of the three examined strategies were only tested by a single study. Beyond the need for more studies testing these strategies, as well as studies testing additional strategies, a major gap in the literature identified after data extraction includes the lack of representation of infants and limited representation of toddlers.

## 4 Discussion

Findings suggest that children are more likely to sleep during childcare when they are provided with a specific time to nap without the ability to engage in other activities, and when providers actively assist in helping them rest. To our knowledge, this is the only systematic review of prior research on structural and behavioral sleep promotion strategies used in childcare settings. The low number of studies meeting our eligibility criteria, despite the relatively lenient parameters employed (i.e., no restrictions on geographical location or publication date), indicate that this is an understudied area of research on behavioral pediatric sleep. Notably, there were significant overlaps in authorship across studies conducted in the United States (*n* = 2) (Field, 1999; Field et al., 1996) and Australia (*n* = 3) (Staton et al., 2015, 2017; Thorpe et al., 2018), which further demonstrates the limited number of researchers conducting work on this topic.

Four out of the six studies identified assessed the effects of mandatory naptime in childcare settings (Fujii et al., 2022; Staton et al., 2015, 2017; Thorpe et al., 2018). Three of these studies were conducted in Australia (Staton et al., 2015, 2017; Thorpe et al., 2018); the other was conducted in Malaysia (Fujii et al., 2022). Given that childcare practices and regulations may vary across states and countries (Benjamin Neelon et al., 2014; Fujii et al., 2022; Fukuda and Sakashita, 2002), lack of geographical diversity poses concerns for external generalizability of the findings. This is especially true because geographic locations may also correspond with differences in cultural napping norms (e.g., napping is more prevalent in Asian countries) (Mindell et al., 2013). In addition, future studies are also needed to specifically assess the influence of different contexts and social norms surrounding naps across different cultures and geographic areas.

The remaining two studies, conducted by the same researcher, assessed the effects of music and massage therapy on children's nap latency in the United States (Field, 1999; Field et al., 1996). In the 1996 study, the authors posited that massage improved children's mood and cooperative behavior, thus reducing nap latency. In the 1999 study, the authors referred to an increase in alpha brain waves, usually associated with a state of relaxation, as a potential mechanism. Indeed, providing a relaxing environment for sleep is generally recommended for childcare settings by keeping a low noise and light level (McGuire, 2012). Notably, these were the oldest articles by publication date (i.e., 1996 and 1999) (Field, 1999; Field et al., 1996). The absence of more recent studies examining behavioral sleep promotion strategies in childcare raises concerns around generalizability and highlights the dearth of research. This represents a critical research gap given that prior studies have demonstrated that there is considerable variability in childcare providers' practices during naptime (Staton et al., 2016; von Ash et al., 2021). For example, variations in provider practices include assisting children in falling asleep and established pre-nap routines (Staton et al., 2016; von Ash et al., 2021). Examining the impact on children's sleep of such variations in provider behavior is essential to develop best practices.

In terms of findings, music and massage therapy may be effective strategies to reduce nap latency, however these findings need to be replicated (Field, 1999; Field et al., 1996). Because each type of intervention was tested by just a single study, conclusions about key aspects of the intervention, such as ideal dose, cannot be made. Nonetheless, the reduction in latency to sleep when children were exposed to either music or massage, despite the interventions not being delivered and tested in a way that allowed integration into a consistent nap routine, is notable. Importantly, since these studies were published, which was much earlier than the rest of the studies included in this review (1996–1999 vs. 2015–2022), a strong body of literature has demonstrated how helpful consistent routines, including around sleep (Kitsaras et al., 2018; Mindell et al., 2015; Mindell and Williamson, 2018), are for children. The music intervention (Field et al., 1996), delivered for just 2 days, was not of sufficient duration to allow for the formation and realized benefits of having a consistent nap routine. The massage intervention (Field, 1999), was delivered over a longer period (i.e., 5 weeks), but just two times per week, creating inconsistency in the routine. Coupling the findings from each study with the existing literature around routine consistency, both types of interventions may be especially beneficial if integrated into a consistent nap routine. In addition, while playing music during naptime is not an uncommon practice in childcare (von Ash et al., 2021), children's sleep may be differentially impacted based on the volume, duration, and type of music played. Notably, while playing music is a relatively non-intensive and low burden intervention, incorporating massage therapy as a standard practice raises questions regarding feasibility and appropriateness. Studies examining the feasibility of providers implementing the aforementioned (and other) sleep promotion strategies are also needed. For example, while some children may benefit from having a provider rub their back at the start of naptime, that could be difficult when there are numerous children per adult. On the other hand, reducing the light level in the room during naptime, is likely more feasible.

Regarding structural sleep promotion strategies, mandatory naptime appeared to be associated with higher incidence and proportion of napping children as well as longer nap duration (Fujii et al., 2022; Staton et al., 2015, 2017; Thorpe et al., 2018). This suggests that more children fall asleep during childcare if they are provided with the opportunity (Fujii et al., 2022; Staton et al., 2015, 2017; Thorpe et al., 2018). However, mandatory naptime was not only associated with sleep during childcare, but also nighttime and total sleep duration in the one longitudinal study (Staton et al., 2015). Specifically, Staton et al. (2015) found an inverse relationship between mandatory naptime and nighttime sleep duration as well as total daily sleep duration (Staton et al., 2015), which was in contrast to findings from the cross-sectional study by Fujii et al. (2022) but consistent with prior research (Acebo et al., 2005; Fukuda and Asaoka, 2004; Jones and Ball, 2013; Ward et al., 2008).

This is important as the goal, especially for older children in childcare (i.e., preschool age children, the large majority of whom cease napping by age 5), should be that children have an opportunity to nap during childcare to help meet their sleep needs, as opposed to forcing them to sleep if their needs are already being met by nighttime sleep (S. Staton et al., 2020). That said, making changes to naptime policies in childcare settings, will likely come with logistical considerations to the larger operations of the childcare facility. For example, there are childcare facilities that take advantage of naptime to also provide their staff with legally mandated breaks (O'Hagan et al., 2025), thus changing the naptime structure may have staffing implications. Furthermore, one qualitative study found that parents may have differing opinions about how naptime is structured in their children's daycare based on how they think naps impact their children's nighttime sleep (Sinclair et al., 2016).

The timing of children's sleep periods throughout the day could have health and developmental implications, given that longer nighttime sleep duration indicates a more advanced phase of sleep consolidation (Staton et al., 2020). Sleep consolidation occurs when children transition from having multiple sleeping periods, to two periods (i.e., naptime and nighttime sleep), to finally one period (i.e., at night) (Staton et al., 2020). More advanced sleep consolidation, marked by longer nighttime sleep, is associated with lower risk of language delay and better outcomes on cognitive measures (Dionne et al., 2011; Lam et al., 2011). Future studies should examine whether there are downstream effects of mandatory naptime on developmental outcomes through negatively affecting sleep consolidation. It is worth noting though that participants in the 2015 Staton et al. study were 4–6 years old and an estimated 80% of children cease napping by age 5 (Staton et al., 2020). It makes sense that mandating naptime for children who do not require a nap to meet their sleep needs could be consequential to nighttime sleep, but this is unlikely the case for those who rely on naps to get sufficient sleep (e.g., younger children and populations with documented sleep disparities). Strengths of the current systematic review include the use of rigorous methods consistent with the PRISMA methodology (Moher et al., 2009). We summarized the current state of the literature on sleep promotion strategies in childcare settings and identified research gaps. However, this review is not without limitations. Firstly, this review was conceptualized out of a previous systematic review. Consequently, there were differences between the registered protocol for the first review and the current review. Specifically, the research questions between the first and the current review differed, because the current review was a follow up to the first review. Further, based on the recommendation we received from a health sciences librarian, an additional database (i.e., CINAHL) was added to the search, and this was not reflected in the registered protocol. Secondly, while we do not expect that we missed many studies, it is possible that additional studies may have been identified if the search was not limited to three databases and articles written in English. Lastly, due to the limited number of studies and variability in interventions, outcomes, and study designs, a meta-analysis could not be performed and synthesis of findings across studies was limited. Given this and that all studies received a quality assessment of “fair,” implying for a need for improvements in methodological rigor, caution is warranted when interpreting specific findings. That said, we see the main take home message from this review as being less about the impact of specific strategies and more about the dearth of research on such an important topic.

## 5 Conclusion

Findings from this systematic review suggest that sleep related practices during childcare likely impact children's sleep outcomes, which was also supported by qualitative research (O'Hagan et al., 2025). For example, providers in one study also reported using similar strategies to help children fall asleep (e.g., music, rubbing backs) (O'Hagan et al., 2025). However, the paucity of research conducted on this topic is highlighted. As there is a well-established body of evidence for how sleep impacts young children's wellbeing (Dionne et al., 2011; Lam et al., 2011; Matricciani et al., 2019; Staton et al., 2020), these findings provide a rationale for further examination of the impact of sleep promotion strategies in childcare settings. Given that young children, especially racial and ethnic minority children, rely on naps to meet their sleep needs, identifying effective sleep promotion strategies in childcare settings may help address sleep disparities. In conclusion, the efficacy of sleep promotion strategies in childcare settings is an important but understudied area of research.

Additional studies using experimental designs and more rigorous methods, such as randomized controlled trials, are highly needed. Moreover, additional aspects of the childcare environment that may be relevant to sleep need to be identified and assessed with regards to their impact on children's sleep outcomes, not only during the day but also throughout 24 h. Examples of these additional aspects include consistency of the naptime schedule, ambient environment (e.g., light and noise), and changes in staffing coverage during naptime. Findings from this line of research can be used to identify areas of need and opportunities to promote sleep health by leveraging the childcare context. Lastly, future studies should prioritize using objective sleep measures, such as actigraphy, and increasing diversity of participants as well as geographic locations.

## References

[B1] Acebo C. Sadeh A. Seifer R. Tzischinsky O. Hafer A. Carskadon M. A. (2005). Sleep/wake patterns derived from activity monitoring and maternal report for healthy 1- to 5-year-old children. Sleep 28, 1568–1577. doi: 10.1093/sleep/28.12.1568 16408417

[B2] Agaronov A. Ash T. Sepulveda M. Taveras E. M. Davison K. K. (2018). Inclusion of sleep promotion in family-based interventions to prevent childhood obesity. Child. Obes. 14, 485–500. doi: 10.1089/chi.2017.0235 30109955 PMC6422003

[B3] Ahn S. Lobo J. M. Logan J. G. Kang H. Kwon Y. Sohn M.-W. (2021). A scoping review of racial/ethnic disparities in sleep. Sleep Med. 81, 169–179. doi: 10.1016/j.sleep.2021.02.027 33713923

[B4] Albakri U. Drotos E. Meertens R. (2021). Sleep health promotion interventions and their effectiveness: an umbrella review. Int. J. Environ. Res. Public Health 18:11. doi: 10.3390/ijerph18115533 PMC8196727 34064108

[B5] Benjamin Neelon S. E. Duffey K. Slining M. M. (2014). Regulations to promote healthy sleep practices in child care. Pediatrics 134, 1167–1174. doi: 10.1542/peds.2014-0578 25384491 PMC4243065

[B6] Cai S. Tham EKH. Xu HY. Fu X. Goh RSM. Gluckman PD. . (2023). Trajectories of reported sleep duration associate with early childhood cognitive development. Sleep 46:zsac264. doi: 10.1093/sleep/zsac264 36355436 PMC9905782

[B7] Chaput JP. Gray CE. Poitras VJ. Carson V. Gruber R. Birken CS. . (2017). Systematic review of the relationships between sleep duration and health indicators in the early years (0–4 years). BMC Public Health 17, 91–107. doi: 10.1186/s12889-017-4850-2 29219078 PMC5773910

[B8] Covidence, (n.d.). Covidence. Covidence. Available online at: https://www.covidence.org/ (Accessed December 5, 2024).

[B9] Crosby B. LeBourgeois M. K. Harsh J. (2005). Racial differences in reported napping and nocturnal sleep in 2- to 8-year-old children. Pediatrics 115, 225–232. doi: 10.1542/peds.2004-0815D 15866856 PMC2987587

[B10] DeFrancisis J. S. Vinarski B. M. Abreu M. Ibrahim J. Hostoffer R. (2025). Efficacy of osteopathic manipulative treatment in creating a difference in pain levels for patients with localized joint pain: a meta-analysis of randomized controlled trials. Cureus 17:e82724. doi: 10.7759/cureus.82724 40406764 PMC12097846

[B11] Dionne G. Touchette E. Forget-Dubois N. Petit D. Tremblay RE. Montplaisir JY. . (2011). Associations between sleep-wake consolidation and language development in early childhood: a longitudinal twin study. Sleep 34, 987–995. doi: 10.5665/SLEEP.1148 21804661 PMC3138173

[B12] Field T. (1999). Music enhances sleep in preschool children. Early Child Dev. Care 150, 65–68. doi: 10.1080/0300443991500106

[B13] Field T. Kilmer T. Hernandez-Reif M. Burman I. (1996). Preschool children's sleep and wake behavior: effects of massage therapy. Early Child Dev. Care. 39–44 doi: 10.1080/0300443961200104

[B14] Fujii M. Hayashi M. Teng C. L. (2022). Nap patterns of children in kindergartens and childcare transit facility: a study in northern peninsular Malaysia. Sleep Sci. 15, 128–134. doi: 10.5935/1984-0063.20220011 35273758 PMC8889960

[B15] Fukuda K. Asaoka S. (2004). Delayed bedtime of nursery school children, caused by the obligatory nap, lasts during the elementary school period. Sleep Biol. Rhythms 2, 129–134. doi: 10.1111/j.1479-8425.2004.00129.x

[B16] Fukuda K. Sakashita Y. (2002). Sleeping pattern of kindergartners and nursery school children: function of daytime nap. Percept. Mot. Skills 94, 219–228. doi: 10.2466/pms.2002.94.1.219 11883566

[B17] Jones C. H. D. Ball H. L. (2013). Napping in English preschool children and the association with parents' attitudes. Sleep Med. 14, 352–358. doi: 10.1016/j.sleep.2012.12.010 23466349

[B18] Kamara J. K. Akombi B. J. Agho K. Renzaho A. M. N. (2018). Resilience to climate-induced disasters and its overall relationship to wellbeing in Southern Africa: a mixed-methods systematic review. Int. J. Environ. Res. Public Health 15:2375. doi: 10.3390/ijerph15112375 30373194 PMC6267582

[B19] Kitsaras G. Goodwin M. Allan J. Kelly M. P. Pretty I. A. (2018). Bedtime routines child wellbeing and development. BMC Public Health 18:386. doi: 10.1186/s12889-018-5290-3 29562892 PMC5861615

[B20] Lam J. C. Mahone E. M. Mason T. Scharf S. M. (2011). The effects of napping on cognitive function in preschoolers. J. Dev. Behav. Pediatr. 32:90. doi: 10.1097/DBP.0b013e318207ecc7 21217402 PMC3095909

[B21] Lupini F. Williamson A. A. (2023). Health disparities in pediatric sleep. Sleep Med. Clin. 18, 225–234. doi: 10.1016/j.jsmc.2023.01.005 37120165 PMC10210975

[B22] Magee L. Goldsmith LP. Chaudhry UAR. Donin AS. Wahlich C. Stovold E. . (2022). Nonpharmacological interventions to lengthen sleep duration in healthy children: a systematic review and meta-analysis. JAMA Pediatr. 176:1084. doi: 10.1001/jamapediatrics.2022.3172 36094530 PMC9468945

[B23] Matricciani L. Paquet C. Galland B. Short M. Olds T. (2019). Children's sleep and health: a meta-review. Sleep Med. Rev. 46, 136–150. doi: 10.1016/j.smrv.2019.04.011 31121414

[B24] McGuire S. (2012). Institute of medicine (IOM) early childhood obesity prevention policies. Washington, DC: the national academies press; 2011. Adv. Nutr. 3, 56–57. doi: 10.3945/an.111.001347 22332102 PMC3262615

[B25] Mindell J. A. Li A. M. Sadeh A. Kwon R. Goh D. Y. T. (2015). Bedtime routines for young children: a dose-dependent association with sleep outcomes. Sleep 38, 717–722. doi: 10.5665/sleep.4662 25325483 PMC4402657

[B26] Mindell J. A. Sadeh A. Kwon R. Goh D. Y. T. (2013). Cross-cultural differences in the sleep of preschool children. Sleep Med. 14, 1283–1289. doi: 10.1016/j.sleep.2013.09.002 24269649

[B27] Mindell J. A. Williamson A. A. (2018). Benefits of a bedtime routine in young children: sleep, development, and beyond. Sleep Med. Rev. 40, 93–108. doi: 10.1016/j.smrv.2017.10.007 29195725 PMC6587181

[B28] Moher D. Liberati A. Tetzlaff J. Altman D. G. PRISMA Group. (2009). Preferred reporting items for systematic reviews and meta-analyses: the PRISMA statement. PLoS Med. 6:e1000097. doi: 10.1371/journal.pmed.1000097 21603045 PMC3090117

[B29] National Association for the Education of Young Children (2021). Progress and peril: Child care at a crossroads. National Association for the Education of Young Children. Available online at: https://www.naeyc.org/sites/default/files/globally-shared/downloads/PDFs/resources/blog/naeyc_july_2021_survey_progressperil_final.pdf (Accessed July 5, 2021).

[B30] National Heart Lung, and Blood Institute. (2021). Study Quality Assessment Tools | NHLBI, NIH. Available online at: https://www.nhlbi.nih.gov/health-topics/study-quality-assessment-tools (Accessed July 13, 2026).

[B31] Nevarez M. D. Rifas-Shiman S. L. Kleinman K. P. Gillman M. W. Taveras E. M. (2010). Associations of early life risk factors with infant sleep duration. Acad. Pediatr. 10, 187–193. doi: 10.1016/j.acap.2010.01.007 20347414 PMC2866807

[B32] O'Hagan B. Raisman N. Chmielewski S. von Ash T. (2025). Childcare provider self-reported practices during naptime: a qualitative study. Child and Youth Care Forum. 501–518. doi: 10.1007/s10566-025-09892-2

[B33] Paruthi S. Brooks LJ. D'Ambrosio C. Hall WA. Kotagal S. Lloyd RM. . (2016). Consensus statement of the american academy of sleep medicine on the recommended amount of sleep for healthy children: methodology and discussion. J. Clin. Sleep Med. 12, 1549–1561. doi: 10.5664/jcsm.6288 27707447 PMC5078711

[B34] Reynaud E. Vecchierini M.-F. Heude B. Charles M.-A. Plancoulaine S. (2018). Sleep and its relation to cognition and behaviour in preschool-aged children of the general population: a systematic review. J. Sleep Res. 27:e12636. doi: 10.1111/jsr.12636 29164715

[B35] Sinclair D. Staton S. Smith S. S. Pattinson C. L. Marriott A. Thorpe K. (2016). What parents want: parent preference regarding sleep for their preschool child when attending early care and education. Sleep Health 2, 12–18. doi: 10.1016/j.sleh.2015.11.002 29073446

[B36] Staton S. Marriott A. Pattinson C. Smith S. Sinclair D. Thorpe K. (2016). Supporting sleep in early care and education: an assessment of observed sleep times using a sleep practices optimality index. Sleep Health 2, 30–34. doi: 10.1016/j.sleh.2015.12.005 29073449

[B37] Staton S. Rankin PS. Harding M. Smith SS. Westwood E. LeBourgeois MK. . (2020). Many naps, one nap, none: a systematic review and meta-analysis of napping patterns in children 0–12 years. Sleep Med. Rev. 50:101247. doi: 10.1016/j.smrv.2019.101247 31862445 PMC9704850

[B38] Staton S. L. Smith S. S. Hurst C. Pattinson C. L. Thorpe K. J. (2017). Mandatory nap times and group napping patterns in child care: an observational study. Behav. Sleep Med. 15, 129–143. doi: 10.1080/15402002.2015.1120199 26751779

[B39] Staton S. L. Smith S. S. Pattinson C. L. Thorpe K. J. (2015). Mandatory naptimes in child care and children's nighttime sleep. J. Dev. Behav. Pediatr. 36:235. doi: 10.1097/DBP.0000000000000157 25853460

[B40] Tandon P. S. Saelens B. E. Christakis D. A. (2015). Active play opportunities at child care. Pediatrics 135, e1425–e1431. doi: 10.1542/peds.2014-2750 25986016 PMC4444799

[B41] Taveras E. M. Rifas-Shiman S. L. Oken E. Gunderson E. P. Gillman M. W. (2008). Short sleep duration in infancy and risk of childhood overweight. Arch. Pediatr. Adolesc. Med. 162, 305–311. doi: 10.1001/archpedi.162.4.305 18391138 PMC2650815

[B42] Thorpe K. J. Pattinson C. L. Smith S. S. Staton S. L. (2018). Mandatory naptimes in childcare do not reduce children's cortisol levels. Sci. Rep. 8:4545. doi: 10.1038/s,41598-018-22555-8 29540702 PMC5852241

[B43] U.S. Department of Education and National Center for Education Statistics. (2021). Early Childhood Program Participation: 2019, (NCES 2020-075REV), Table 1. National Center for Education Statistics. Available online at: https://nces.ed.gov/fastfacts/display.asp?id=4 (Accessed July 13, 2026).

[B44] Vaughn B. E. Elmore-Staton L. Shin N. El-Sheikh M. (2015). Sleep as a support for social competence, peer relations, and cognitive functioning in preschool children. Behav. Sleep Med. 13, 92–106. doi: 10.1080/15402002.2013.845778 24527839

[B45] von Ash T. O'hagan B. Gupta A. Deokule N. Josephson A. Chmielewski S. . (2025). It's time to put the nap in NAPSACC: a systematic review demonstrating the impact of childcare on sleep outcomes in early childhood. Child. Obes. 21, 255–272. doi: 10.1089/chi.2024.0384 40228049 PMC13159755

[B46] von Ash T. Risica P. M. Tovar A. Gans K. M. (2021). An observational assessment of sleep environments in family child care homes. Int. J. Epidemiol. Public Health Res. 1.

[B47] Ward T. M. Gay C. Anders T. F. Alkon A. Lee K. A. (2008). Sleep and napping patterns in 3-to-5-year old children attending full-day childcare centers. J. Pediatr. Psychol. 33, 666–672. doi: 10.1093/jpepsy/jsm102 17956928

[B48] Zhang Z. Adamo K. B. Ogden N. Goldfield G. S. Okely A. D. Kuzik N. . (2021). Longitudinal correlates of sleep duration in young children. Sleep Med. 78, 128–134. doi: 10.1016/j.sleep.2020.12.023 33429288

